# Managing Yao Syndrome: A Case of Beneficial Treatment with Upadacitinib and Leflunomide

**DOI:** 10.31138/mjr.261224.erh

**Published:** 2025-07-15

**Authors:** Georges El Hasbani, John M Davis

**Affiliations:** Division of Rheumatology, Mayo Clinic, Rochester, MN, United States

**Keywords:** Yao syndrome, NOD2 mutations, abdominal bloating, refractory cases, JAK inhibitors

## Abstract

Yao syndrome, an autoinflammatory disease associated with specific NOD2 gene variants, presents with a wide range of symptoms, including fever, dermatitic rashes, polyarthritis, abdominal pain/bloating, and sicca symptoms. The initial clinical manifestation remains widely variable. The first-line treatment options remain unknown due to limited knowledge of the pathophysiology and the absence of robust literature. We report a case of a 36-year-old woman diagnosed with Yao syndrome, presenting with postprandial bloating, followed by polyarthralgia and low-grade fever. After experiencing reactions to treatments, including anaphylaxis secondary to anakinra and canakinumab, the patient showed improvement with upadacitinib and leflunomide, resulting in better control of her symptoms.

## INTRODUCTION

Systemic autoinflammatory diseases are a group of periodic inflammatory diseases without detectable auto-antibodies or antigen-specific T lymphocytes.^1^ Multiple autoinflammatory diseases have been linked to gene variants. The nucleotide-binding oligomerisation domain-containing protein-2 (NOD2) is a cytosolic NOD-like receptor (NLR) that was discovered in 2001,^2^ and its variants have been linked to multiple autoinflammatory diseases, such as Blau syndrome^3^ or Yao syndrome.^4^

Yao syndrome may present in various ways, but it is characterised by periodic fever, dermatitic rashes, polyarthritis, gastrointestinal (GI) symptoms, and sicca symptoms.^4^ The presenting symptom is commonly fever, arthralgia, or diffuse erythematous rash.^5^ Less commonly, abdominal pain or diarrhoea might signify a Yao syndrome flare^6^. Since Yao syndrome is still an emerging disease with limited understanding of its pathophysiology and scarce data from large randomised controlled trials, the first-line management options remain uncertain. Glucocorticoids and sulfasalazine have been considered treatment options, while interleukin (IL)-1/IL-6 inhibitors are reserved for cases of refractory disease activity.^4^

Herein, we present the case of a 36-year-old woman initially evaluated for postprandial bloating, which was later associated with polyarthralgia, fatigue, and low-grade fever. She experienced treatment failure with methotrexate and leflunomide, as well as an anaphylactic reaction to anakinra and canakinumab. She was subsequently maintained on upadacitinib and leflunomide, achieving better control of disease activity.

## CASE PRESENTATION

**Figure 1.**
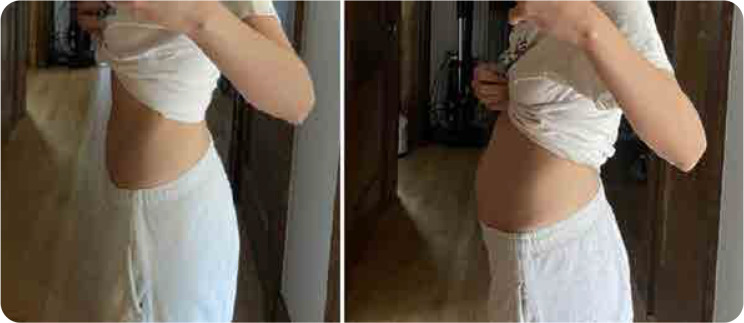
Pre-prandial (A) and post-prandial (B) abdomen.

A 36-year-old Brazilian woman with a past medical history of eosinophilic asthma presented with episodes of postprandial bloating (**Figure 1A–B**). She had no family history of autoinflammatory or autoimmune diseases. An endoscopy was negative for any acute pathologies. Her symptoms were followed by fatigue and polyarthralgia. These symptoms were associated with episodes of a few days of low-grade fever. She had a previous diagnosis of seronegative rheumatoid arthritis and was being treated with subcutaneous methotrexate and leflunomide, which provided partial improvement of her polyarthralgia. She also had a history of anaphylactic reaction to adalimumab.

On physical examination, she exhibited symmetric articular tenderness without evidence of clinical synovitis. Inflammatory markers, including ESR and CRP, were negative, even during the low-grade fever episodes. Serologies, including ANA and ENA panels, were also negative. HLA-B27 gene analysis was negative. A nuclear medicine joint scan showed symmetrical uptake in the bilateral shoulders, hips, and DIP joints, while an MRI of the pelvis was negative for sacroiliitis.

NOD2 complete gene analysis was positive for the R702W and IVS8+158 variants (double heterozygous), but the full 116-gene autoinflammatory panel via rapid DNA sequencing was otherwise negative. She was diagnosed with Yao syndrome. Consequently, she was switched to the IL-1 inhibitor anakinra 100 mg subcutaneous daily, along with continued methotrexate 15 mg weekly and leflunomide 20 mg daily. However, she developed ongoing hives after starting anakinra, although the medication reduced the frequency of postprandial bloating and polyarthralgia. Therefore, anakinra was discontinued. Subsequently, liver function tests increased, leading to the suspension of methotrexate. Canakinumab 4 mg/kg every 4 weeks was then attempted, but the patient experienced an anaphylactic reaction, resulting in its discontinuation. Upadacitinib 15 mg daily, in combination with leflunomide 20 mg daily, was then tried. A 6-month follow-up revealed a decrease in the frequency of postprandial bloating, inflammatory arthritis symptoms, and low-grade fever. She continued to require a few courses of oral corticosteroids for control of Yao syndrome flares. She had three episodes of a few days of low-grade fever and inflammatory arthritis in a span of six months compared to a flare every month prior to initiating upadacitinib.

## DISCUSSION

The most common presenting symptoms of Yao syndrome remain variable. Fever may occur in up to 81.8% of patients, although the frequency of different patterns may vary.^7^ On the other hand, arthritis may occur in up to 95.5% of cases.^7^ Abdominal bloating has been reported to occur in 58%,^8^ while gastrointestinal symptoms in general can occur in 80% of patients.^9^ In addition, inflammatory markers can be normal in approximately 50% of the cases.^9^

The best first-line treatment option for Yao syndrome remains unknown due to a limited understanding of its pathophysiology and the absence of large randomised clinical trials due to the heterogeneity and infrequency of this disease.^10^ While glucocorticoids seem to provide a good control of flares,^9^ cases of refractory Yao syndrome have been reported. Interleukin-1 inhibitors, anakinra and canakinumab, and interleukin-6 inhibitors, sarilumab and tocilizumab, have been tried for these cases with variable outcomes (**Table 1**). Notably, 64.9% (37 out of 57 patients) in one relatively large cohort of Yao syndrome responded to IL-1 inhibitors, unspecified whether anakinra or canakinumab.^11^

**Table 1.** Effectiveness of interleukin-1 and interleukin-6 inhibitors on patients with Yao syndrome.

**Agent**	**Study**	**No. of Patients**	**Outcome**
**Anakinra**	Yao and Shen^4^	2	Partial response for both patients
	DeMaio et al.^18^	1	No response
	Williamson et al.^19^	3	2 Full response 1 Partial response
**Canakinumab**	Yao and Shen^4^	2	Partial response for both patients
	Ahmad and Kilian^20^	1	Full response
	Yao^21^	7	Full response for all patients
	Brailsford et al.^22^	1	Full response
	Zhang et al.^23^	1	Full response
**Tocilizumab**	Williamson et al.^19^	5	2 Partial response 3 full response
	Brailsford et al. ^22^	1	Failure
	Yao and Shen^4^	2	Partial response
	Zhang et al.^23^	1	Failure
	Yao and Shen^4^	1	Full response
	Nomani et al.^8^	4	Partial response
**Sarilumab**	Williamson et al.^19^	1	Failure

Janus Kinase inhibitors (JAK inhibitors, JAKi) disrupt signal transduction in the Janus Kinase-Signal Transducer and Activator of Transcription (JAK-STAT) pathway, leading to effective suppression of downstream cytokine signaling.^12^ These agents have been utilised in multiple autoinflammatory diseases, such as axial spondyloarthropathies^13^ and psoriatic arthritis.^14^ Less commonly, JAK inhibitors have been used in Yao syndrome, resulting in improved symptoms in 3 out of 4 of the patients treated.^9^ Certain JAK inhibitors, such as tofacitinib, have been used in Familial Mediterranean Fever with good clinical response^15^. Similarly, JAK inhibitors have been used in a total of 35 patients with adult-onset Still’s disease who were refractory to first-line treatment. Of these, 17 (48.6%) patients showed complete remission.^16^ Upadacitinib has been reported to be used in anti-TNF-refractory macular edema associated with Behçet’s uveitis.^17^ However, such data is based on a limited number of patients. In addition, upadacitinib has been found to be rapidly effective and safe in inflammatory bowel disease,^18^ which is also associated with NOD2 susceptibility variants.^19^

Leflunomide has long been used in autoinflammatory diseases and works by inhibiting pyrimidine metabolism, leading to a reduction in Th1 inflammatory responses.^20^ The combination of JAK-inhibitors and leflunomide has shown effectiveness in rheumatoid arthritis^21^ but has not been tested for NOD2-associated autoinflammatory diseases.

## CONCLUSION

In the presented case, the patient with Yao syndrome showed improvement in postprandial bloating and inflammatory arthritis symptoms after treatment with upadacitinib and leflunomide. Despite initial challenges with anakinra and canakinumab, due to adverse reactions, the combination of JAK inhibitors and leflunomide appeared to be a beneficial therapeutic option. These findings suggest that further investigation into JAK inhibitors for NOD2-associated autoinflammatory diseases like Yao syndrome may be warranted.

## DISCLAIMER

No part of this manuscript, including the text and photos, are copied or published elsewhere in whole or in part.

## INFORMED CONSENT

Written informed consent obtained from the patient.

## CONFLICT OF INTEREST

Dr. Davis has received a research grant from Pfizer. Dr. El Hasbani has no conflicts of interest.
